# Silent Threats: A Narrative Review of Urinary Bladder Cancer in Dogs and Cats—Epidemiology and Risk Factors

**DOI:** 10.3390/ani16020217

**Published:** 2026-01-12

**Authors:** Isabel Pires, Rita Files

**Affiliations:** 1 Department of Veterinary Sciences, University of Trás-os-Montes and Alto Douro, 5000-801 Vila Real, Portugal; ipires@utad.pt; 2 Associate Laboratory for Animal and Veterinary Sciences (AL4AnimalS), Animal and Veterinary Research Centre (CECAV), University of Trás-os-Montes and Alto Douro, 5000-801 Vila Real, Portugal

**Keywords:** canine, feline, environmental exposure, urothelial carcinoma, urinary bladder

## Abstract

Urinary bladder neoplasms are clinically relevant in dogs and cats and are also common in humans, all of which may share exposure to environmental factors that influence disease risk. In Veterinary Medicine, however, their etiological determinants remain poorly defined. Urinary bladder neoplasia range from non-invasive lesions limited to the mucosa to invasive forms that infiltrate the muscular layer, which are more aggressive and metastatic. In dogs, invasive urothelial carcinoma (UC) represents the most frequently diagnosed type, while in cats, it is less common but displays similar biological behavior. Hematuria and dysuria are the predominant clinical signs, and although urinary bladder cancer accounts for only a small proportion of canine neoplasms, it is associated with considerable morbidity and mortality. Several risk factors have been identified, including breed, sex, age, obesity, diet, neuter status, and environmental exposures. Female dogs, especially Terrier breeds, are more susceptible, whereas in cats, males and short-haired animals are more often affected. Contact with insecticides, herbicides, and antiparasitic products is a recognized risk factor in dogs, although this association has not been consistently demonstrated in cats. Neutering and obesity appear to increase risk in dogs, and dietary patterns may offer protection, with regular vegetable consumption linked to a reduced incidence. Understanding these determinants is essential to improve early detection, guide preventive measures, and strengthen comparative oncology research.

## Simple Summary

Urinary bladder cancer is a highly aggressive disease in pets, yet its risk factors remain poorly explored. Early diagnosis is difficult because clinical signs often resemble those of common urinary tract infections. Current evidence highlights several potential contributors, including age, sex, neutering status, obesity, breed-related genetic predisposition, environmental exposures, tobacco smoke, certain flea control products, and diet. These factors may act independently or interact to increase susceptibility. A clearer understanding of these risks is essential to improve disease recognition, support earlier diagnosis, and reduce animals’ exposure to preventable predisposing factors.

## 1. Introduction

Urinary bladder cancer is one of the most prevalent malignant neoplasms of the urinary tract in both humans and companion animals. Although various histological subtypes, including adenocarcinoma, sarcoma, and squamous cell carcinoma, may occur, urothelial carcinoma (UC), also known as Transitional Cell Carcinoma, accounts for nearly 90% of cases and is characterized by progressive infiltration of the basement membrane, lamina propria, and muscular layers [1,2].

In humans, urinary bladder cancer development is strongly influenced by environmental exposures to chemical agents and air pollution, as well as by genetic susceptibility and behavioral habits, including smoking, alcohol consumption, and diet [3,4]. Although risk factors in companion animals are not yet fully defined, growing evidence demonstrates significant parallels between species. Dogs and cats share the same domestic environments as their owners, and several determinants linked to human urinary bladder cancer, such as age, genetic predisposition, and specific environmental exposures, have also been reported in animals [4,5,6,7].

Breed-associated susceptibility in dogs further underscores the role of hereditary and environmental influences; specific purebred groups exhibit markedly higher disease frequencies. These findings have increased interest among veterinarians, breeders, and owners in understanding risk profiles and disease prevention in predisposed populations [4,5,6].

Despite these advances, considerable gaps persist in our understanding of UC in companion animals. The actual burden of environmental carcinogens, the interaction between genetic susceptibility and metabolic factors, and the specific contribution of domestic exposures (pesticides, herbicides, industrial residues, household chemicals, tobacco smoke) remain poorly defined. Moreover, animals may serve as early sentinels of environmental risk, reflecting exposures difficult to quantify in humans, a perspective that reinforces the importance of veterinary epidemiology in identifying emerging hazards [4,7,8].

The aggressive biological behavior of urothelial carcinoma and its frequent diagnosis at advanced stages have important therapeutic implications in companion animals. Recent advances in diagnostic imaging, cytology, molecular markers, and minimally invasive sampling techniques have improved early detection and staging; however, effective treatment remains challenging. In dogs, therapeutic options include surgery, chemotherapy, and radiation therapy, although long-term survival remains limited. Electrochemotherapy has been explored in a limited number of cases, but its clinical efficacy and safety in companion animals require further investigation [9,10]. Prognosis is influenced by both local complications, such as urinary tract obstruction, and intrinsic tumor characteristics, including proliferative activity, invasiveness, and metastatic potential [11]. In cats, reported treatment options include cystectomy, nonsteroidal anti-inflammatory drugs, chemotherapy, and radiation therapy [12]; however, access to these modalities is variable, and standardized therapeutic or screening guidelines are still lacking.

Given the clinical relevance of UC and the limitations of current epidemiological knowledge in veterinary medicine, there remains a need for an updated, integrative synthesis focused on companion animals that brings together available evidence on risk factors for this neoplasm. Although comparative and species-specific studies addressing different aspects of UC have been published, information on risk determinants remains largely fragmented, and an integrated discussion that compiles these factors within a unified veterinary and comparative oncology perspective, encompassing both dogs and cats, remains limited.

Therefore, this narrative review aims to contribute to this field by integrating the currently available evidence on the epidemiology and risk factors of UC in companion animals, with particular emphasis on modifiable determinants, species-specific differences, and existing knowledge gaps, especially in cats. We aim to provide an updated overview to inform future diagnostic, preventive, and research strategies in veterinary oncology.

## 2. Materials and Methods

This narrative review was conducted to compile and synthesize current evidence on the epidemiology and risk factors of urothelial carcinoma in dogs and cats. An initial exploratory search of the human urinary bladder cancer literature was performed to identify well-established risk factors and to guide the selection of relevant determinants for evaluation in the veterinary context.

A structured literature search was subsequently conducted in the PubMed and Google Scholar databases. The last search was performed on 20 November 2025. The final search strings used in PubMed included combinations such as (“urothelial carcinoma” OR “transitional cell carcinoma” OR “urinary bladder cancer”) AND (dog OR canine) AND (“risk factors” OR pesticides OR herbicides OR diet OR obesity OR neutering), and analogous combinations replacing dog/canine with cat/feline. Similar search strategies were applied in Google Scholar.

Additional cross-species searches were performed using terms such as “urothelial carcinoma animals”, “environmental carcinogens urinary bladder cancer”, “diet urinary bladder cancer animals”, and “comparative oncology urinary bladder cancer”. The reference lists of selected articles were manually screened to identify further relevant publications.

Inclusion criteria comprised peer-reviewed original articles and reviews published between 1989 and 2025 that addressed urinary bladder urothelial carcinoma in dogs and/or cats and evaluated at least one epidemiological or risk factor (e.g., age, sex, breed, obesity, neutering, environmental or dietary exposures). Studies primarily focused on clinical presentation or histopathology were also included when they provided data relevant to disease frequency, distribution, biological behavior, or interpretation of risk determinants. Studies exclusively describing diagnostic techniques, therapeutic approaches, or isolated case reports without epidemiological or risk-related information were excluded, as were conference abstracts, editorials, letters to the editor, and other non-peer-reviewed sources.

Titles and abstracts were first screened for relevance, followed by full-text assessment of potentially eligible articles.

## 3. Urinary Bladder Cancer in Companion Animals

**Figure 1:**
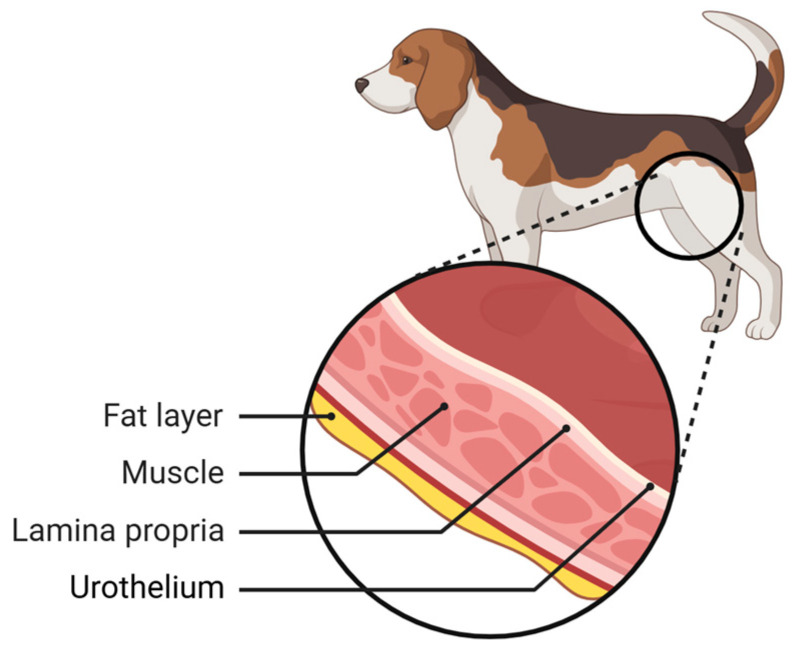
Representation of the layers of the bladder wall, adapted from [18].

Urothelial carcinoma is the most common malignant neoplasm of the urinary tract in dogs, although it is rarely reported in cats [13]. This neoplasia originates from the epithelial cells that line the urinary bladder [14,15], the urothelium, one of the bladder layers (Figure 1). The urothelium (or transitional epithelium) is formed by a thick layer of stratified, non-squamous, relatively homogeneous cells with “umbrella” cells on the surface. About 90% of urinary bladder neoplasms are urothelial carcinomas [16,17,18]. UC also occurs in the ureter, urethra, prostatic ducts, and renal pelvis [14,15].

Urinary bladder cancer affects tens of thousands of dogs annually worldwide, accounting for approximately 2% of all canine cancer cases [19,20]. The survival time for these tumors in dogs is approximately 1 to 2 years (Table 1) [21,22].

In contrast, the incidence of UC in cats is much lower, accounting for approximately 0.38–0.56% of all feline malignancies, possibly due to differences in exposure to risk factors, as discussed later. The survival time of a cat with UC is approximately 261 days (Table 1) [12].

In dogs, this neoplasm can be classified into two main categories: non-muscle-invasive urinary bladder cancer and muscle-invasive urinary bladder cancer. Most newly diagnosed canine patients have muscle-invasive bladder cancer (90%), which is clinically relevant because this form allows for less conservative and bladder-sparing treatment options [23,24,25,26]. Urinary bladder neoplasia can also be staged based on the characteristics of the primary tumor (T), regional lymph node involvement (N), and the presence of distant metastases (M), as in human neoplasia. However, the staging classification of UC differs between dogs and humans. In dogs, T1 tumors are superficial and do not invade the lamina propria, while in humans, T1 tumors already invade it. Thus, invasive cancer in dogs is classified as T2 or T3: T2 invades the muscle, and T3 reaches adjacent organs. In humans, T2 invades the muscle, T3 invades the fat layer, and T4 invades neighboring organs. Therefore, 78% of canine UC cases correspond to stage T2 (invasion of the bladder wall) and 20% to stage T3 (invasion of neighboring organs) [12,19,22,27,28,29].

In cats, no standardized staging system has been established, and classification is usually extrapolated from canine or human TNM schemes, reflecting the scarcity of feline-specific epidemiological and pathological data [30].

The vesical trigone is the most common site of involvement in dogs, with frequent bladder wall thickening and papillary lesions that can cause partial or total obstruction of the urinary tract. The extension to the urethra has been reported in 56% of dogs and to the prostate in 29% of males [31]. The location of urinary bladder cancer in cats appears to be more variable than in dogs. In cats, the trigonal region does not seem to be the predominant site, with a reported prevalence of 27.1% (32 of 118 cases), and tumors may arise in various areas of the urinary bladder outside the trigone (Table 1) [12].

Clinically, both dogs and cats commonly present signs of lower urinary tract disease, including stranguria, hematuria, and polyuria. In advanced stages, local progression may result in urinary obstruction, painful abdominal distension, and, less frequently, bladder rupture. UC often invades the urinary bladder wall, and at the time of diagnosis, approximately 20% of dogs already present with metastatic disease, which is associated with a poorer prognosis [12,19,27,28]. Longitudinal studies indicate that up to 58% of affected dogs develop distant metastases over the course of the disease. Metastatic spread in dogs most often involves regional lymph nodes, bones, lungs, and liver [15,20,31].

In cats, metastasis rates range from 12.7% to 20% of cases. Lung and lymph node metastases are the most commonly reported (Table 1), but sporadic reports of spread to other organs, such as the large intestine, eyeball, and abdominal wall, have been reported. In some instances, abdominal wall involvement has been attributed to tumor seeding along the needle track during cystocentesis procedures used to obtain diagnostic samples [32,33,34,35].

**Figure 2:**
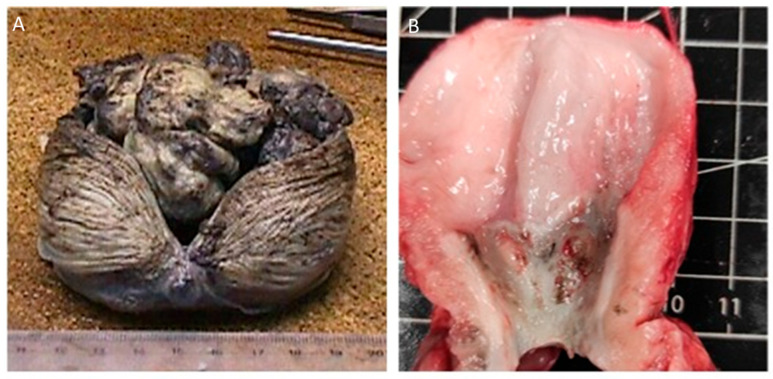
Gross morphology of canine urothelial carcinoma. (**A**) Papillary urothelial carcinoma displaying a multilobulated, exophytic growth pattern. (**B**) Nonpapillary urothelial carcinoma with a solid, irregular, and infiltrative mass in the vesical trigone, author’s own image.

Grossly, urothelial carcinomas in both species may present as papillary or nonpapillary lesions, often with a solid, infiltrative appearance [30]. When uncontrolled, the primary lesion can obstruct the urinary tract, resulting in death [12,19,27,28]. Figure 2 shows examples of the gross morphology of the canine urinary bladder UC.

A comparative summary of the epidemiological and clinical characteristics of urothelial carcinoma in dogs and cats is presented in Table 1.

**Table 1:** Epidemiological and clinical characteristics of urothelial carcinoma in dogs and cats.

Characteristics	Dogs	Cats
Prevalence	~2% of all canine cancers [19,20]	~0.38–0.56% of all feline cancers [30,33]
Predominant Location	Vesical trigone [31]	Outside the trigone [12]
Clinical signs	Stranguria, hematuria, polyuria; advanced: obstruction, bladder rupture [15,31]	Stranguria, hematuria, polyuria; advanced: obstruction, bladder rupture [15,31]
Gross morphology	Papillary or nonpapillary, solid/infiltrative	Papillary or nonpapillary, solid/infiltrative
Grade/invasiveness	Mostly high-grade invasive; 78% invade the bladder wall; 20% invade adjacent organs [19,27,28]	Data limited; high-grade tumors reported, variable location [30]
Metastases	Lymph nodes, bones, lungs, liver [20]	Lungs and lymph nodes [34,35]
Metastases at diagnosis	~20% [19,27,28]	12.7–20% [34,35]
% cases that develop metastases	58% [19,27,28]	Data limited
Median survival	Variable; 1–2 years [21,22]	~261 days [12]
Prognosis	Poor [19,27,28]	Poor [12]

## 4. Risk Factors

Several factors can contribute to the development of UC, ranging from hereditary genetic predisposition to environmental influences [19]. Among the main risk factors identified are exposure to older formulations of flea control products, contact with certain lawn chemicals, obesity, neutering, breed, sex, and age [19,22].

### 4.1. Genetic Predisposition and Breed Susceptibility

**Figure 3:**
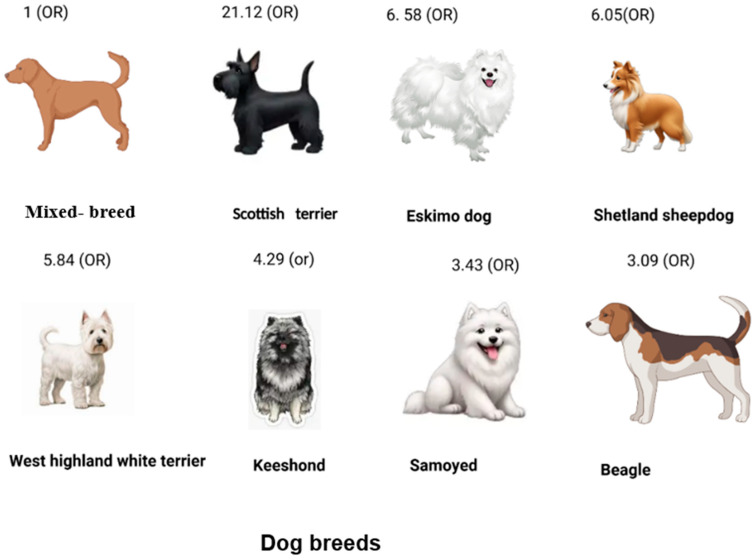
Odds ratio (OR) for urothelial carcinoma in different dog breeds compared with mixed-breed dogs, adapted from [31].

Certain diseases show marked breed-associated patterns in companion animals, often attributable to underlying morphological or genetic characteristics. In other instances, however, particular breeds exhibit increased disease prevalence without a clearly defined etiological basis [31,36,37]. As shown in Figure 3, Scottish Terriers have the highest risk of developing urinary bladder cancer (OR = 21.12). Increased odds are also observed in Eskimo Dogs (OR = 6.58), Shetland Sheepdogs (OR = 6.05), West Highland White Terriers (OR = 5.84), Keeshonds (OR = 4.29), Samoyeds (OR = 3.43), and Beagles (OR = 3.09), compared with mixed-breed dogs used as the reference group (Table 2) [22,37,38,39,40,41]

These pronounced breed predispositions support the hypothesis that genetic factors contribute substantially to disease development, although environmental influences, such as diet and exposure to phenoxy herbicides, may further modulate risk. The genetic homogeneity of purebred dogs provides a valuable framework for identifying genomic alterations conserved across evolution, some of which may be relevant to human disease. Such information may therefore carry important clinical implications in both Veterinary and Human Medicine [42].

In cats, no definitive genetic association with urothelial cancer has been identified. However, several studies report a higher prevalence in short-haired individuals. Breeds such as the British Shorthair Blue, Domestic Longhair, Devon Rex, and Domestic Shorthair also appear to be over-represented (Table 2) [30,33,43,44].

**Table 2:** Dog and cat breeds with a higher predisposition to urothelial carcinoma (UC) of the urinary bladder.

Species	Breed	Relative Risk
Dogs	Scottish Terrier [37,38,39,40,41]	~21× higher risk
	West Highland White Terrier, Shetland Sheepdog, Beagle [37,38,39,40,41]	~3–6× higher risk
	Wire-Haired Fox Terrier [37,38,39,40,41]	~3–5× higher risk
Cats	British Shorthair Blue, Domestic Longhair, Devon Rex, Domestic Shorthair [30,33,43,44]	Over-represented; no definitive genetic link
	Short-haired cats [30,33,43,44]	Higher prevalence reported

### 4.2. Sex and Age

Sex plays a crucial role in the incidence, prognosis, and mortality of various types of cancer. Biological differences between males and females directly interfere with neoplasm behavior [45].

In the specific case of urinary bladder cancer, the literature describes a higher prevalence in females in dogs (Table 3) [19,38]. Among possible explanations is that female dogs urinate less frequently than males and have a higher body fat percentage. As many carcinogenic chemicals tend to be stored and concentrated in adipose tissue, females may accumulate more carcinogens over time [39,41,46,47]. However, in humans, men consistently show a higher risk of developing urinary bladder cancer. Research indicates that males are two to four times more likely than females to develop this disease, even when accounting for established risk factors such as smoking, occupational exposures, and infections [48].

Unlike in dogs, clinical studies in cats indicate a higher proportion of males affected by lower urinary tract diseases and bladder neoplasms (Table 3). Male cats are more susceptible to urethral obstruction and urinary retention, conditions that can promote prolonged urinary stasis and chronic inflammation of the urinary bladder, potentially contributing to the development of urinary bladder cancer [33,34,49,50].

Age is also an important determinant, as the disease occurs predominantly in older animals, with most affected dogs and cats presenting between 9 and 11 years of age (Table 3) [31,50,51,52].

### 4.3. Environmental Factors (e.g., Herbicides, Pesticides)

The urinary bladder is continuously exposed to environmental toxins and inflammatory stimuli, making it particularly vulnerable to carcinogenic insults. Among the most relevant preventable risk factors for urinary bladder cancer is exposure to chemical carcinogens. Pesticides used in domestic, agricultural, and industrial settings, including herbicides, insecticides, and fungicides, represent an essential source of such exposure. Many of these compounds and their metabolites are excreted in urine, supporting the urogenital contact hypothesis, in which carcinogens dissolved in urine directly interact with and transform urinary bladder epithelial cells [40,53].

Epidemiological studies have linked urinary bladder cancer in dogs to household pesticide use, residence near industrial areas, and consumption of drinking water with elevated concentrations of total trihalomethanes [37,54,55]. Dogs exposed to pesticides have an increased risk of developing urinary tract neoplasms and may act as sentinel species for environmental hazards affecting humans. Several studies demonstrate that dogs with a history of exposure to lawn herbicides and insecticides have significantly higher odds of developing urinary bladder cancer, particularly those exposed to recently treated or water-saturated areas [16,56,57].

**Figure 4:**
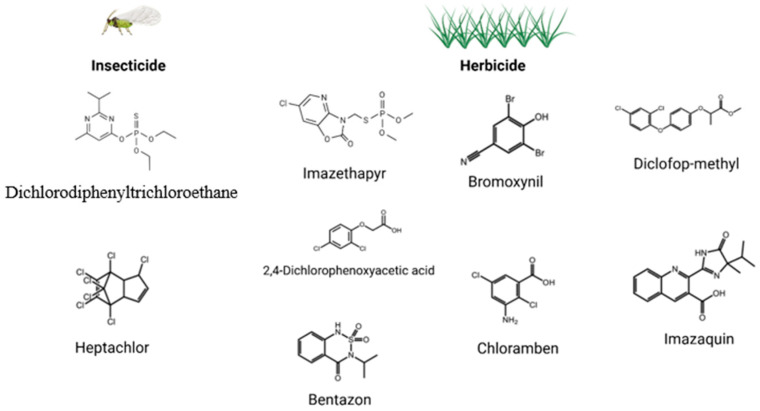
Pesticides and herbicides associated with an increased risk of urothelial carcinoma, adapted from [54,59].

Among the herbicides most frequently implicated are phenoxy acids (e.g., 2,4-Dichlorophenoxyacetic Acid (2,4-D), Mecoprop (MCPP), and 2-Methyl-4-chlorophenoxyacetic Acid (MCPA), benzoic acids (e.g., dicamba), and organophosphate compounds such as glyphosate. These products have been detected in canine urine and linked to neoplasia development (Figure 4). Their detection in dogs without known direct exposure further suggests environmental contamination or drift from neighboring treated areas [58,59].

Flea and tick baths use compounds such as organophosphates, carbamates, pyrethrins, and pyrethroids, often combined with synergists such as piperonyl butoxide. Up to 96% of their formulations may consist of “inert” ingredients, including petroleum distillates, aromatic solvents, polyethers, and xylene, as well as potentially toxic substances such as acrolein and inorganic arsenic. These products have been associated with an increased risk of UC in dogs [38,60,61]; however, comparable data in cats are currently lacking.

Pesticides may contribute to carcinogenesis through multiple molecular mechanisms, including the modulation of pathways involved in tumor progression and metastasis, such as epithelial–mesenchymal transition (EMT). Epithelial–mesenchymal transition (EMT) is a reversible process that decreases cell adhesion, causes loss of epithelial polarization, and confers on tumoral cells the ability to migrate, invade, resist apoptosis and senescence, and to exhibit stem cell characteristics, while favoring immunosuppression and contributing to metastasis. It is characterized by reduced E-cadherin and increased MMP-9, both of which are associated with unfavorable clinical outcomes. Several pesticides can intensify EMT by altering gene expression and modifying cell signaling pathways. E-cadherin, a protein essential for cell–cell adhesion, when lost, releases β-catenin, which acts as a coactivator of transcription factors and regulates gene expression. The organophosphate malathion, used in mosquito control, alters the actin cytoskeleton and decreases E-cadherin and β-catenin in mammary cancer cells. Low-dose exposures also increase the activity of Rho and Rac1 GTPases, promoting migration and invasion. Although there is no direct evidence, malathion can accumulate in the urinary bladder after thermal exposure [58,62,63].

Activation of the Transforming Growth Factor β (TGF-β) pathway is an important stimulus for EMT. Herbicides containing arsenic, such as Dimethylarsinic acid (DMA), the main urinary metabolite of inorganic Arsenic, elevate TGF-β levels and induce EMT by regulating HER2. Paraquat also increases TGF-β in lung cells and in animal models, leading to changes similar to EMT and fibrogenesis. Pesticide exposure has a similar effect in other tissues: endosulfan modulates the TGF-β/Smad pathway in human renal mesangial cells; occupational exposure increases TGF-β1 in mammary cancer cells; and the HGF/c-Met/TGF-β pathway, associated with urinary bladder oncogenesis, can be activated by pesticides, contributing to metastasis and representing a promising therapeutic target through antibodies and small-molecule inhibitors [58,62,63].

**Figure 5:**
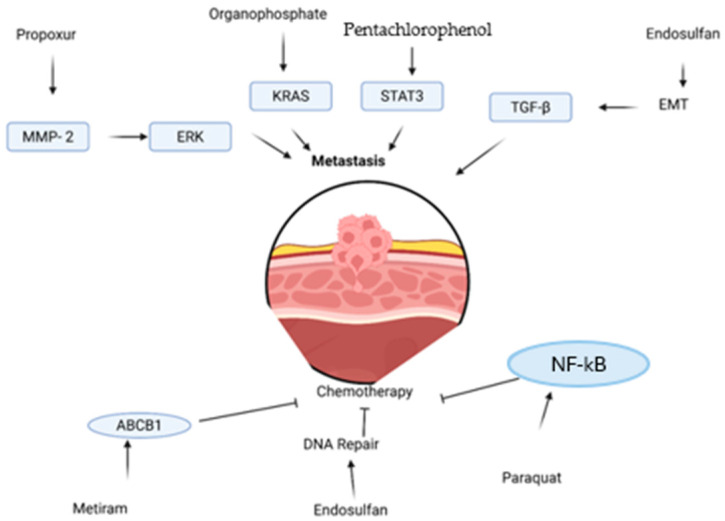
Pathways involved in pesticide-induced urinary bladder cancer progression and metastasis. MMP-2 (Matrix metalloproteinase-2), ERK (Extracellular signal-regulated kinase), KRAS (Kirsten rat sarcoma viral oncogene homolog), STAT3 (Signal transducer and activator of transcription 3), TGF-β (Transforming growth factor beta), EMT (Epithelial–mesenchymal transition), ABCB1 (ATP-binding cassette sub-family B member 1, P-glycoprotein), NF-κB (Nuclear factor kappa B), PCP (Pentachlorophenol). The diagram illustrates how different pesticides (Propoxur, Organophosphate, PCP, Endosulfan, Paraquat, Metiram) influence molecular pathways associated with metastasis, EMT, DNA repair, and chemotherapy resistance in urinary bladder cancer cells, adapted from [63].

Organophosphates also increase the expression of proteins associated with EMT, such as vimentin, axin, and slug, while chlorpyrifos elevates MMP2 and vimentin, promoting cell invasion. Another relevant regulator is STAT3, which is constitutively activated in various neoplasms and also by pesticides and is conventionally involved in invasion, migration, and angiogenesis. STAT3 phosphorylation is caused by IL-6, which stimulates VEGF and, consequently, angiogenesis. Pesticides such as pentachlorophenol increase IL-6 and chemokine production, and elevated IL-6 levels in the urinary bladder are associated with advanced clinical stages, higher recurrence, and lower survival. Therefore, chronic exposure to pesticides promotes molecular and signaling changes that favor EMT, increase invasiveness, and affect metastasis in urinary bladder cancer, with potential therapeutic targets in the TGF-β, HGF/c-Met, and STAT3 pathways. (Figure 5) [58,62,63].

These findings suggest that pesticides not only increase the risk of urinary bladder cancer but may also promote metastasis, highlighting the importance of investigating these pathways as potential therapeutic targets [58].

### 4.4. Dietary Influences

Diet is another factor that may modulate urinary bladder cancer risk in both humans and companion animals. Certain dietary behaviors can reduce exposure to carcinogens or interfere with carcinogenic pathways, thereby preventing or delaying the development of neoplasms. Adequate fluid intake and the regular consumption of fruits and vegetables appear particularly relevant. In humans, the association between vegetable intake and the risk of transitional cell carcinoma is somewhat conflicting; however, several epidemiological studies suggest a 20% to 60% reduction in risk among individuals with higher vegetable consumption [61,64].

In dogs, dietary habits also appear to influence the occurrence of UC. Among Scottish Terriers, a breed with a markedly elevated predisposition, consumption of any vegetable at least 3 times per week was associated with a 70% reduction in UC risk. Intake of yellow–orange vegetables or leafy green vegetables at the same frequency conferred additional benefit, with risk reductions of approximately 70% and 90%, respectively. In this study, commercial dry food constituted the predominant baseline diet, being consumed daily by more than 95% of both affected and control dogs, and no significant association was observed between the general category of commercial diet and UC risk. This suggests that the protective effect was associated with the inclusion of vegetables rather than with the type of commercial food itself [61,65]. Although commercial dry and wet foods represent the most common diets in companion animals, direct evidence linking specific categories of commercial pet food to urinary bladder cancer risk remains scarce and warrants further investigation [61,65].

To date, epidemiological data on cats specifically addressing the role of diet, including commercial wet and dry foods, in urinary bladder cancer risk are lacking.

### 4.5. Other Potential Risk Factors (Obesity, Hormonal Status, Castration or Sterilization, Environmental Tobacco Smoke, Swimming in Pools)

**Figure 6:**
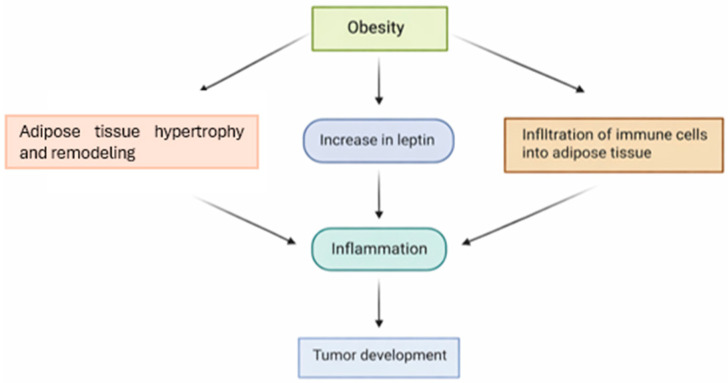
Obesity can initially cause chronic inflammation, which can subsequently promote neoplasia, adapted from [67].

Obesity compromises the well-being and health of animals. Obese dogs exhibit reduced life expectancy and quality of life. They are more prone to developing comorbidities such as orthopedic disorders, cardiovascular and respiratory diseases, insulin resistance, and certain types of neoplasms. In this context, obesity has also been identified as a risk factor for urinary bladder neoplasia. Excess white adipose tissue results in dysregulated adipokine production and increased secretion of pro-inflammatory cytokines. Among these adipokines, leptin plays a particularly relevant role, as its elevated concentrations in obese animals contribute to chronic inflammation, enhance cell proliferation, promote angiogenesis, and may facilitate tumor progression (Figure 6). This chronic low-grade inflammatory state, often accompanied by insulin resistance, creates a biological environment conducive to neoplasia initiation and progression and has been associated with a higher incidence and greater malignancy of several cancer types (Figure 6) [48,62,66,67,68,69]. Consequently, dogs exposed to carcinogenic environmental factors and concurrently affected by metabolic disorders, such as obesity, may present an even greater likelihood of developing cancer, which can ultimately impair the effectiveness of therapeutic interventions [48,62,68,69].

Surgical sterilization, performed by ovariectomy, ovariohysterectomy, or castration, is widely used to control overpopulation and prevent reproductive diseases such as mammary cancer and prostatic hyperplasia. Removal of the gonads alters hormonal balance, eliminating negative feedback from sex hormones and raising luteinizing hormone (LH) levels to 30 times higher than in intact dogs. LH acts on various tissues, including the urinary bladder and urethra, and can influence cell division and promote carcinogenesis [31,68,69].

Studies show that neutered dogs have a higher incidence of urothelial cancer compared to intact animals [31,68,69]. These procedures have been identified as a risk factor for both sexes. In an epidemiological sample of 260 dogs with UC, sterilization was associated with a significantly higher risk of developing the disease (odds ratio ~4.57), indicating that neutered dogs were about 4.6 times more likely to develop UC than unneutered dogs [65]. In cats, the available literature suggests an association between gonadectomy and an increased incidence of urothelial carcinoma; however, the evidence is limited and less consistent than in dogs, largely due to the low prevalence of the disease and the scarcity of robust epidemiological studies [65].

In addition, gonadectomy can increase appetite, reduce metabolic rate, and promote obesity, as well as cause changes in the urinary tract, such as incontinence, and musculoskeletal disorders. The combination of these hormonal and metabolic factors may contribute to the development of transitional cell carcinoma of the urinary bladder in neutered dogs [31,68,69].

Environmental tobacco smoke contains detectable concentrations of several carcinogens, including arylamines, which are strongly associated with the development of UC in humans; approximately half of human UC cases are attributed to smoking. Although earlier veterinary studies did not demonstrate a clear association between canine UC and smoking within the household, a recent investigation was the first to evaluate the relationship between owner-reported tobacco use and urinary cotinine levels in dogs. The study documented measurable cotinine concentrations in dogs exposed to environmental tobacco smoke, with levels increasing proportionally to the number of cigarettes smoked indoors in the preceding 24 h, indicating effective absorption of toxic compounds. Because dogs and cats live in domestic environments with humans and are exposed through inhalation, dermal contact, and oral ingestion during grooming, they may serve as biological sentinels for environmental risks relevant to young children, who have close contact with contaminated surfaces such as floors, carpets, furniture, countertops, toys, and pet bedding [70,71,72].

In addition, the study identified swimming in pools as a significant risk factor for the development of UC in dogs, possibly due to chlorination byproducts such as trihalomethanes and bromoform, which are known mutagens associated with UC in humans. Although this finding is consistent with epidemiological studies in humans, confirmation in larger canine populations is necessary, considering potential related variables [71].

## 5. Final Considerations

UC in companion animals represents a multifactorial disease shaped by genetic, metabolic, and environmental determinants. Although progress has been made in identifying risk factors, particularly in dogs, significant gaps persist in understanding their relative contributions and interactions. The influence of pesticides, herbicides, and other environmental contaminants deserves special attention, given the shared exposure of humans and animals within domestic environments.

However, this review has several limitations. The available evidence is heterogeneous and largely based on retrospective and observational studies, which limits the strength of causal inferences. Potential publication bias and reliance on owner-reported exposure histories in many studies may further affect the robustness of the described associations. Moreover, most data derive from canine populations. At the same time, epidemiological evidence in cats remains scarce. It is mainly limited to factors such as age, sex, breed, and reproductive status, necessitating cautious extrapolation from canine or human studies in some sections. Differences in study design, exposure assessment, geographic context, and outcome definitions across reports hinder direct comparisons between risk factors. In addition, for several environmental and dietary determinants, the evidence is based on a limited number of studies, some of which are relatively dated, and mechanistic data in companion animals remain sparse. Nevertheless, the available findings already highlight relevant patterns that may inform preventive actions and help identify priority areas for future research.

Strengthening prevention requires coordinated efforts from veterinarians and urologists, who play complementary roles in recognizing early risk indicators and guiding owners toward evidence-based risk reduction. Greater awareness of modifiable factors, including obesity, environmental exposure, and avoidable chemical contact, is essential.

Early diagnosis is equally critical, considering the aggressive behavior, high metastatic potential, and significant clinical impact of UC in both species. Advances in diagnostic techniques and staging methods offer promising avenues for more precise and individualized patient assessment, ultimately improving clinical outcomes.

Future research should prioritize large-scale, prospective epidemiological studies, molecular profiling of neoplasms, and the development of sensitive biomarkers for early detection. Integrated comparative oncology approaches involving human and companion animal populations may facilitate the identification of shared carcinogenic pathways, common molecular signatures, and geographic areas of increased environmental risk. Such strategies could enable the mapping of high-risk regions, support targeted public health and veterinary interventions, and strengthen the role of companion animals as sentinels for environmental carcinogenesis.

A deeper understanding of environmental carcinogenesis, breed-associated susceptibility, and cross-species neoplasia biology will advance more targeted preventive strategies and translational research, with a positive impact on prognosis and quality of life for affected animals and potential relevance for human urinary bladder cancer prevention and management.

## Figures and Tables

**Table 3:** Age and sex as risk factors for urothelial carcinoma (UC) of the urinary bladder in dogs and cats.

Feature	Dogs	Cats
Sex predominance	Females [19,38]	Males [33,34,49,50]
Age	9–11 years [31]	9–11 years [50,51]

## Data Availability

No new data were created.

## Abbreviations

The following abbreviations are used in this manuscript:

UC	Urothelial carcinoma
WHO	World Health Organization
OR	Odds ratio
2,4-D	2,4-Dichlorophenoxyacetic Acid
MCPA	2-Methyl-4-Chlorophenoxyacetic Acid
MCPP	Mecoprop
c-MET	Receptor tyrosine kinase
EMT	Epithelial–Mesenchymal Transition
HGF	Hepatocyte Growth Factor
IL-6	Interleukin 6
STAT3	Signal Transducer and Activator of Transcription
TGF-β	Transforming Growth Factor beta
MMP-9	Matrix Metalloproteinase 9
LH	Luteinizing Hormone
CRP	C-Reactive Protein
ROS	Reactive Oxygen Species
HRAS	Harvey rat sarcoma virus oncogene 1
CEL	Carboxyl Ester Lipase gene
TNF-α	Tumor Necrosis Factor alpha
